# Effectiveness of a pathway-driven eHealth-based integrated care model (PEICM) for community-based hypertension management in China: study protocol for a randomized controlled trial

**DOI:** 10.1186/s13063-021-05020-2

**Published:** 2021-01-22

**Authors:** Zheyu Wang, Chengling Li, Wencai Huang, Yan Chen, Yuqiong Li, Libin Huang, Mei Zhang, Dan Wu, Li Wang, Huilong Duan, Jiye An, Ning Deng

**Affiliations:** 1 College of Biomedical Engineering and Instrument Science, The Ministry of Education Key Laboratory of Biomedical Engineering, Zhejiang University, Hangzhou, 310027 China; 2 The First People’s Hospital of Yibin, Yibin, China; 3 ZICT Technology Co.,Ltd., Shenzhen, China

**Keywords:** Hypertension management, Integrated care, Pathway, Community, Telehealth, Study protocol, Randomized controlled trial

## Abstract

**Background:**

The prevalence of hypertension is high and increasing in China in recent years. The treatment and control of hypertension calls for long-term management beyond hospital, which is hard to implement in traditional care settings. Integrated care combined with information technology can promote high-quality healthcare services across the life-course. However, few studies have applied a customized integrated care model in community-based hypertension management in China, catering to the emerging “three-manager” mode. This study aims to identify the effectiveness of a pathway-driven eHealth-based integrated model that implemented as a full-featured telehealth system to facilitate standardized management of hypertension in China.

**Methods:**

The trial has been designed as a 1-year, non-blinded superiority trial with two parallel groups. A total of 402 hypertensive patients who meet the eligibility criteria will be recruited and randomized with a 1:1 allocation. All the participants will receive a mobile device for self-management, which is a part of our telehealth system. Participants in the control group will only use the device for BP measurement and receive regular follow-ups from care providers according to the guidelines. Participants in the intervention group will gain full access to the system and receive intervention based on the proposed model (a well-designed coordinated care pathway consisting of 9 tasks). Outcomes will be measured mainly on three occasions (at inclusion, at 6 months, and at 12 months). The primary outcome is mean change in systolic blood pressure over a 12-month period. Secondary outcomes include changes in diastolic blood pressure, biochemical indexes related to hypertension, lifestyles, self-management adherence, and hypertension awareness, as well as work efficiency of care providers.

**Discussion:**

This study aims to investigate whether a pathway-driven eHealth-based integrated care model based on the “three-manager” mode will improve hypertension control in China. Success of the model would help improve the quality of present community-based management procedures and benefit more patients with uncontrolled hypertension.

**Trial registration:**

Chinese Clinical Trial Registry ChiCTR1900027645. Registered on November 22, 2019.

**Supplementary Information:**

The online version contains supplementary material available at 10.1186/s13063-021-05020-2.

## Administrative information

Note: the numbers in curly brackets in this protocol refer to Additional file 1: SPIRIT checklist item numbers. The order of the items has been modified to group similar items (see http://www.equator-network.org/reporting-guidelines/spirit-2013-statement-defining-standard-protocol-items-for-clinical-trials/).

**Table:** 

Title {1}	Effectiveness of a pathway-driveneHealth-based integrated care model (PEICM) for community-based hypertension management in China: study protocol for a randomized controlled trial
Trial registration {2a and 2b}.	Chinese Clinical Trial Registry ChiCTR1900027645, registered on November 22, 2019.
Protocol version {3}	Issue Date: 7 Nov 2019 Protocol Version: Original This manuscript details the protocol on the original version approved on 7 November 2019.
Funding {4}	This study was supported by the National Key Research and Development Program of China (No.2017YFC0114105, No.2018YFC0910503, No.2017YFB1002301), the Key Research and Development Program of Ningxia Hui Autonomous of China (No. 2018BFG02009).
Author details {5a}	^1^College of Biomedical Engineering and Instrument Science, The Ministry of Education Key Laboratory of Biomedical Engineering, Zhejiang University, Hangzhou, China ^2^The First People’s Hospital of Yibin, Yibin, China ^3^ZICT Technology Co., Ltd., Shenzhen, China
Name and contact information for the trial sponsor {5b}	Trial Sponsor: The National Key Research and Development Program of China & The Key Research and Development Program of Ningxia Hui Autonomous of China Contact name: Haoming Li Email: dengn@zju.edu.cn
Role of sponsor {5c}	The funding body had no role in study design and will not have any role during collection, management, analysis and interpretation of data; writing of the manuscript; and the decision to submit results.

## Introduction

### Background and rationale {6a}

Hypertension is a major risk factor for cardiovascular disease and affects more than 20% of the adult population in China, according to the latest national survey [1]. The treatment and control of hypertension calls for long-term patient self-management along with supervision and intervention from doctors [2]. However, hypertension management within the traditional care settings is disconnected and time-consuming, which cannot meet long-term care needs of patients [3]. Integrated care is proposed as an approach to transform health services to meet these challenges [4]. In an integrated care setting, health services are delivered by a coordinated multidisciplinary team of providers, aiming to promote the comprehensive delivery of quality services across the life-course [5].

The advent of information technology facilitates the delivery of integrated care services and has demonstrated potential to improve the outcome of hypertension management [6–8]. The importance of information technology in integrated care can be interpreted from different scales. From an individual scale, care planning is a central approach of integrated care, which aims to deliver more personalized and targeted care by creating shared care plans. The care plans clearly articulate the role of each provider and patient in the care process [9]. The use of information technology is considered to be crucial to facilitate the development of shared care plans [4]. From a group scale, the chronic care model (CCM) is one of the most studied integrated care models [10–13]. The key to the success of CCM has proved to be the bidirectional communication within multidisciplinary teams and the provision of continuous self-management support to patients [14]. Information technology plays a key role in achieving the above goals, which has been described as a complete eHealth-based feedback loop in the eHealth Enhanced Chronic Care Model (eCCM) [15]. To conclude, information technology is essential to implement effective integrated care for hypertensive patients.

In China, hypertension management is currently delivered based on communities, mainly conducted by general practitioners (GPs) [16]. In response to the government policy, physicians and case managers (CMs) are gradually participating in the community-based hypertension management to form a coordinated multidisciplinary team called “three-manager” [17]. Similar to other countries, CMs in China are mainly composed of nurses, working under supervision of GPs [18, 19]. Although the “three-manager” mode has been carried out in some provinces, in practice there still exist some problems. One is that current management guidelines do not clearly specify the responsibility of each role in the “three-manager” mode. Another one is that the unbalanced allocation of medical resources in China brings to the difference in abilities of care providers [20, 21]. GPs and CMs in rural areas can hardly perform comprehensive management following the guidelines. These problems result in a significant gap between standards of care and medical practice in hypertension management [22, 23].

### Objectives {7}

To combat the existing challenges of hypertension management in China, we established a pathway-driveneHealth-based integrated care model (PEICM) for the “three-manager” mode, focusing on specifying the responsibility of involved roles and providing a standardized and executable care pathway for long-term management. Based on PEICM, a telehealth system has been designed and implemented. This paper describes a two-arm randomized controlled trial to evaluate the effectiveness of PEICM through the use of the system.

### Trial design {8}

This 1-year study is designed as a randomized, controlled, and non-blinded superiority trial with two parallel groups and repeated measurements at 3 time points (inclusion, at 6 and 12 months). Randomization will be performed as block randomization with a 1:1 allocation. We used the Additional file 1: SPIRIT checklist [24] and the Additional file 2: TIDieR checklist [25] when writing our manuscript.

## Methods: participants, interventions, and outcomes

### Study setting {9}

The trial will be conducted in Yibin city, Sichuan province, China. Yibin is a midsize city located in the joint region of Sichuan, Yunnan, and Guizhou province. Given its population, location, economy, and living standard of people, Yibin can represent the central region of China to a certain extent [26]. Therefore, hypertensive patients in Yibin are representative in terms of demographic characteristics and technology acceptance. In this trial, the participating physicians (cardiologists) are recruited from the First People’s Hospital of Yibin, which is a tertiary hospital with four major districts (A, B, C, and D). A well-equipped health management center (HMC) is established in B district cooperating with ZICT Technology Co., Ltd. (ZICT). The patient enrolment and outcome measurements will be performed in the HMC. The participating GPs and CMs are from 3 community health centers near the First People’s Hospital of Yibin.

### Eligibility criteria {10}

The inclusion criteria are as follows: (1) 18–80 years old, (2) hypertension diagnosis with uncontrolled blood pressure (BP) for at least 1 year, (3) fluent in Mandarin or Sichuan dialect, (4) able to use the system fluently after instruction, (5) mainly visit the hospital where the trial is hold, and (6) have no walking disability. The exclusion criteria are as follows: (1) participate in another trial within 4 weeks before this study, (2) suffer from mental health problems, (3) suffer from cancer and have received chemotherapy or radiotherapy within half a year before this study, (4) suffer from myocardial infarction, (5) suffer from other serious diseases with natural course shorter than 1 year, (6) have no home internet access, and (7) pregnant or prepare for pregnancy.

### Who will take informed consent? {26a}

All patients eligible for the trial will be invited to HMC in B district to attend the enrolment session. CMs will apprise them of the information regarding the study and promise that their personal information will only be accessed by the investigators. The patients who agree to participate in the study will be asked to sign informed consent forms before allocation.

### Additional consent provisions for collection and use of participant data and biological specimens {26b}

The informed consent has included participants’ permission to the collection of blood and urine samples, which will be analyzed in the current study. No ancillary study is planned.

## Interventions

### Explanation for the choice of comparators {6b}

Management guidelines are considered to be the most convincing evidence to guide the management process. The effectiveness of applying the hypertension management guidelines to primary care in China has been tested [27]. Moreover, the care pathway used in our integrated model is mainly extracted from the guidelines. Therefore, we choose guideline-based management as a usual care intervention for the control group.

### Intervention description {11a}

Participants in the control group will receive interventions based on the 2018 Chinese Guidelines for Prevention and Treatment of Hypertension [16]. The guidelines emphasize 4 key components of the management in primary care: hierarchical long-termfollow-up, health education, self-management support, and two-way referral. In this trial, a guideline-based management procedure is as follows: first, patients will be classified into 2 levels according to whether the patients reach target BP, and then, GPs will conduct long-termfollow-up on patients. For level 1 patients, the follow-up frequency is once every 3 months and the follow-up contents focus on complying with current therapy; for level 2 patients, the follow-up frequency is once every 2–4 weeks and the follow-up contents focus on adjusting current therapy to improve BP control. Health education and self-management support will be involved during the follow-up. In addition, a two-way referral channel will be opened to provide further treatment.

**Fig. 1:**
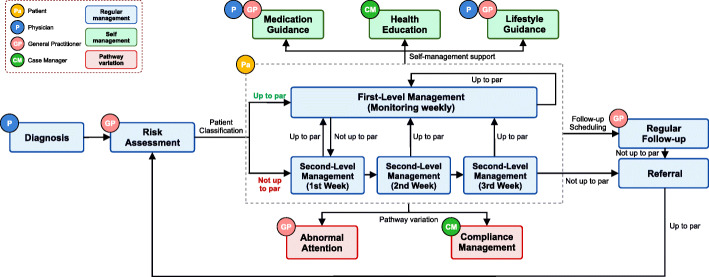
Diagram of PEICM

Participants in the intervention group will receive interventions based on PEICM, as shown in Fig. 1. The model involves 4 roles in the management: physicians, GPs, CMs, and patients. The responsibility of each role is specified through a well-designed care pathway. The care pathway is initialized based on the guidelines and detailed by several experienced physicians. Nine common tasks are defined in the pathway for hypertension management, which are as follows: diagnosis, risk assessment, hierarchical management, medication guidance, lifestyle guidance, regular follow-up, abnormal condition intervention, health education, and compliance management. The tasks will be generated as executable plans, including doctor intervention plans and patient self-management plans.

The 9 tasks can be grouped into 3 parts: the tasks for regular management, the tasks for pathway variation, and the tasks for self-management. The tasks for regular management include 4 tasks: diagnosis, risk assessment, hierarchical management, and regular follow-up. When a patient is diagnosed with hypertension, he/she will enter the pathway. A cardiovascular risk assessment will firstly be conducted to divide patients into 2 management levels: level 1 for patients in low or middle risk while level 2 for patients in high risk. The management plan for level 1 and level 2 is different. In addition, patients’ risk will be reassessed per week based on their self-monitoring data to dynamically change their management levels. Care providers need to perform regular follow-ups for patients, and the follow-up schedule will also be dynamically adjusted based on the management level. Once a patient is in level 2 for over 3 weeks, a two-way referral channel will be opened. The tasks for pathway variation include 2 tasks: abnormal condition intervention and compliance management. When patients’ self-monitoring data is abnormal, care providers will immediately be informed and conduct the appropriate intervention; when patients’ compliance is low, care providers need to perform an extra follow-up to learn the reason for low compliance and try to improve it. The tasks for self-management include 3 tasks: medication guidance, lifestyle guidance, and health education. Medication guidance aims to provide plans about drug prescription while lifestyle guidance aims to provide plans mainly for diet and physical activity. Health education aims to offer health knowledge to patients, in order to improve their hypertension awareness thus improving their self-management ability.

According to the pathway, the responsibility of each role is specified as follows: physicians are responsible for diagnosis and formulating the self-management plans; GPs are responsible for regular follow-ups, plan adjustment, and abnormal condition intervention; CMs are responsible for health education and compliance management. Patients need to follow self-management plans and perform self-monitoring to receive timely intervention from care providers.

**Fig. 2:**
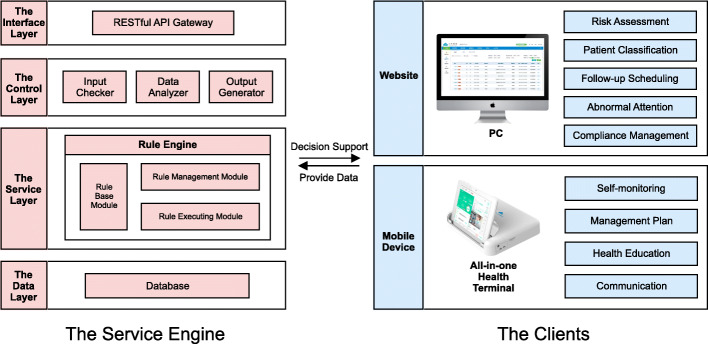
System architecture

Based on PEICM, a telehealth system has been implemented, which consists of 2 parts: clients for care providers and patients, and a service engine for data storage and pathway execution (Fig. 2). For care providers, the client is in the form of a website that can be accessed on their computers in the hospital. Care providers use the client to supervise patients and conduct the intervention. For patients, the client is in the form of an all-in-one health terminal. The terminal is a combination of an Android tablet with a series of wearable devices (integrating measurement of ECG, heart rate, blood pressure, blood glucose, blood oxygen saturation, and body temperature), which was developed by ZICT (Shenzhen, China). Data measured by the terminal will be automatically uploaded and displayed on the website. In addition, patients can use the tablet to check their self-management plans, read health knowledge, and communicate with care providers. The functional design of the tablet application drew on experience from our previous work [28]. The service engine is a web service deployed on the server, consisting of 4 layers: a data layer, a service layer, a control layer, and an interface layer. The engine will be responsible for saving data from clients and providing decision support based on the pathway.

In this trial, all the interventions will be conducted through the system. The intervention group will gain full access to the system and manage their hypertension under the guidance of the model. The control group will only gain partial access to the system (only for BP measurement) and receive care as usual. Table 1 summarizes all the interventions for each group.

**Table 1:** All the interventions for the 2 groups

Intervention category	Execution method in the control group	Execution method in the intervention group
Diagnosis	By physicians (before the trial)	By physicians (before the trial)
Risk assessment	No execution	Conducted by GPs with the assistance of the system; reassessed based on self-monitoring data per week
Hierarchical management	Executed by the system based on patients’ BP values; only adjusted after regular follow-up; no management plans for patients	Executed by the system based on patients’ risk level per week; provide executable management plans to patients
Regular follow-up	Performed by GPs once every 3 months for level 1 patients and once every 2–4 weeks for level 2 patients; no dynamic adjustment	Performed by GPs once every 3 months for level 1 patients and once every 2 weeks for level 2 patients; dynamically adjusted based on patients’ management level
Abnormal condition intervention	No execution	Conducted by GPs; abnormal condition is informed by the system
Compliance management	No execution	Conducted by CMs; compliance is calculated by the system per week
Medication guidance	Provided by GPs during regular follow-up	Provided by physicians or GPs through the system anytime
Lifestyle guidance	Provided by GPs during regular follow-up	Provided by physicians or GPs through the system anytime
Health education	Provided by GPs during regular follow-up	Conducted by CMs through the system, mainly in the form of online materials

### Criteria for discontinuing or modifying allocated interventions {11b}

The assigned intervention will only be discontinued in response to participant request. No modification of the interventions is planned during the trial.

### Strategies to improve adherence to interventions {11c}

In this trial, adherence to interventions mainly refers to patients’ self-management adherence. For the control group, patients will be reminded to follow their self-management regimes during each regular follow-up. For the intervention group, compliance management is defined as a part of the pathway and patient compliance will be calculated by the system per week based on their self-monitoring data. Care providers will not only remind patients in regular follow-ups, but also perform extra follow-ups to improve low adherence.

### Relevant concomitant care permitted or prohibited during the trial {11d}

No concomitant care or interventions are permitted during the trial.

### Provisions for post-trial care {30}

This is not applicable. No post-trial care is planned.

### Outcomes {12}

The primary outcome of the trial is changes in mean systolic blood pressure (SBP) from baseline (T0) to 12 months (T2). The key secondary outcomes are changes in mean diastolic blood pressure (DBP) and changes in biochemical indexes related to hypertension from T0 to T2. The other secondary outcomes include changes in lifestyles, self-management adherence, and hypertension awareness from T0 to T1 (6 months) and T2. In addition, work efficiency of care providers will be assessed at T2 to validate the model from a care provider perspective.

### Participant timeline {13}

**Fig. 3:**
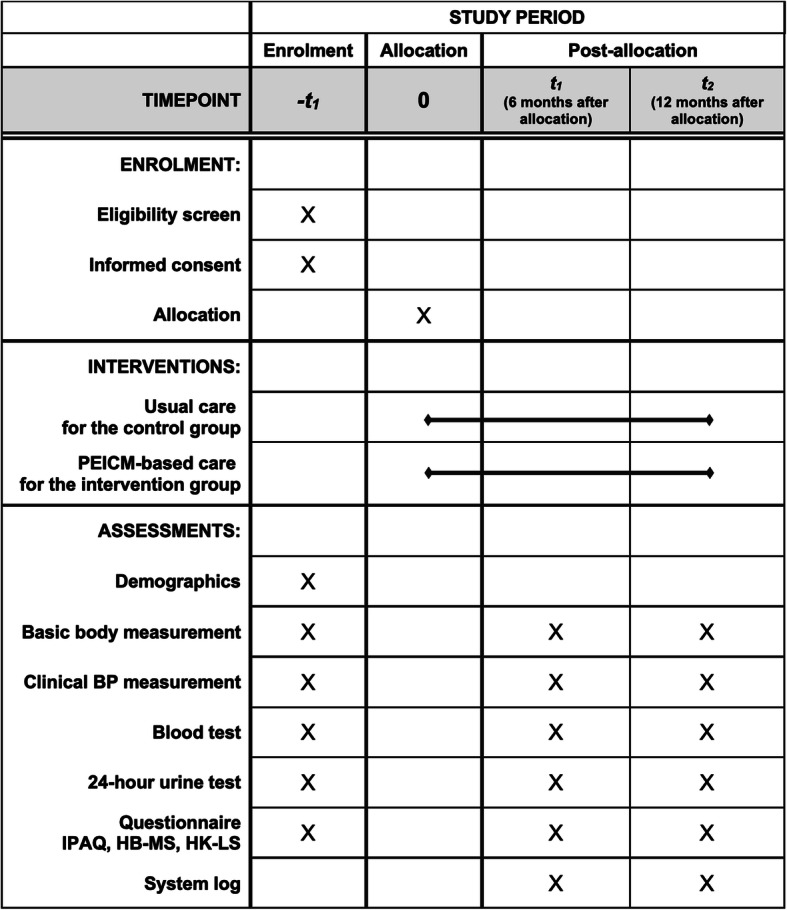
SPIRIT figure

**Fig. 4:**
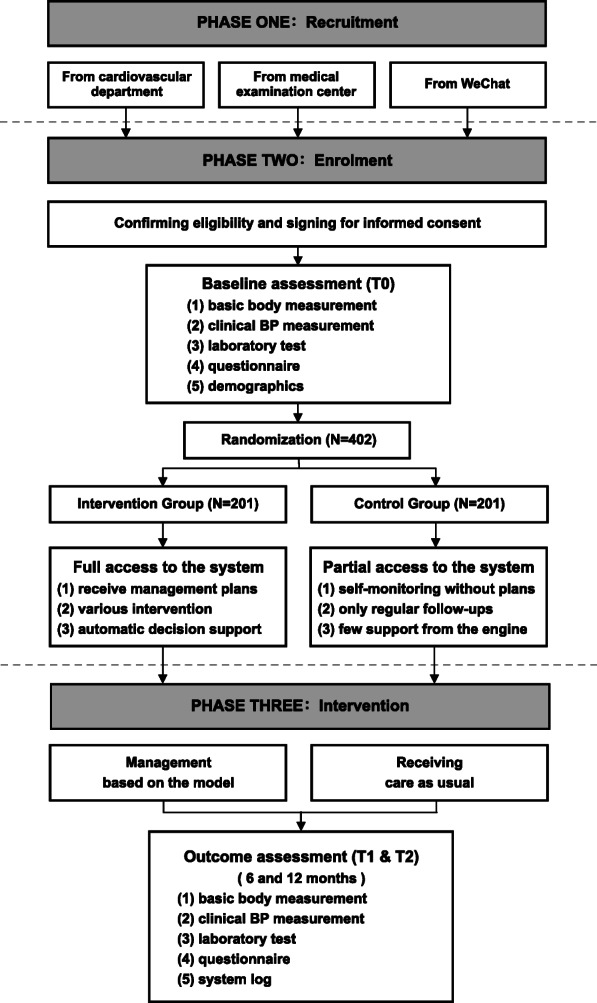
Timeline diagram

The SPIRIT figure is presented in Fig. 3, which provides an overview of the schedule of enrolment, interventions, and assessments. Figure 4 shows the timeline diagram.

### Sample size {14}

The power calculation is based on the primary outcome measure and aims for a mean systolic blood pressure variation of 5 mmHg with a standard deviation of 16.5 mmHg (the effect size is based on a previous study [29]). Assuming an α of 5%, and power of 0.80, 171 patients per group are required at the end of the trial (342 in total). Considering an attrition percentage of 15%, our aim is to include a minimum of 402 patients at baseline.

### Recruitment {15}

Patients are recruited mainly in 3 ways: (1) review discharged patients’ list of the cardiovascular department, (2) review patient list provided by the medical examination center, and (3) recruit patients through physicians’ social media (for example, the functional module Moments in WeChat). CMs will call the potentially eligible patients to confirm the eligibility, then invite them to attend the enrolment session in HMC.

## Assignment of interventions: allocation

### Sequence generation {16a}

One investigator will generate the randomized allocation sequence using SPSS version 23.0, which allocates 50% of the participants to either group.

### Concealment mechanism {16b}

The allocation sequence is concealed in opaque envelopes from all investigators (except for the investigator who generates the sequence) and care providers until the interventions are assigned.

### Implementation {16c}

All patients who give written consent for participation will be enrolled. CMs will guide the enrolment session. First, each patient will be directed to the baseline assessment, including demographics, basic body measurement, clinical BP measurement, laboratory test, and questionnaires. Then, each patient will be randomly assigned an envelope, inside which is a printed random number with the corresponding group. CMs will open the envelope and find the treatment condition to be conducted for this patient. CMs then give the information about treatment allocation to the patient. After that, each group will be guided to different rooms, and CMs will instruct the use of the system to the control group and intervention group, respectively.

## Assignment of interventions: blinding

### Who will be blinded {17a}

Due to the nature of the intervention, this study is an open-label trial to all participants and care providers. However, in order to reduce the level of bias, the statisticians involved in the data analysis will be blinded to the group allocation.

### Procedure for unblinding if needed {17b}

This is not applicable. The trial is a non-blinded trial.

## Data collection and management

### Plans for assessment and collection of outcomes {18a}

Data collection will be mainly conducted at T0, T1, and T2. Table 2 shows the complete outcome measures and their collecting methods. The details are described in the following sections.

**Table 2:** The complete outcome measures and their collecting methods

Outcome measure		Collecting method
Systolic blood pressure		Clinic BP measurement
Diastolic blood pressure		Clinic BP measurement
Biochemical indexes	Blood urea nitrogen (BUN)	Laboratory test (blood test)
	Creatinine (Cr)	Laboratory test (blood test)
	Alanine transaminase (ALT)	Laboratory test (blood test)
	Aspartate transaminase (AST)	Laboratory test (blood test)
	Uric acid (UA)	Laboratory test (blood test)
	Low-density lipoprotein (LDL)	Laboratory test (blood test)
	High-density lipoprotein (HDL)	Laboratory test (blood test)
	Blood glucose (BG)	Laboratory test (blood test)
	Total cholesterol (TC)	Laboratory test (blood test)
	Triglyceride (TG)	Laboratory test (blood test)
Lifestyles	Obesity (BMI and WHR)	Basic body measurement
	Physical activity	Questionnaire (IPAQ)
	Diet (24-h urine sodium and potassium)	Laboratory test (24-h urine test)
Self-management adherence	Medication adherence	Questionnaire (HB-MAS)
	Self-monitoring adherence	System log
Hypertension awareness		Questionnaire (HK-LS)
Work efficiency of care providers	Frequency of follow-up in a day	System log
	Handling time for a follow-up request	System log

#### Blood pressure

Blood pressure (BP) measurement can be categorized into 2 types: clinic and out-of-office BP measurement [16]. Clinic BP measurement is performed by GPs or CMs under standard conditions and unified guidelines. Out-of-office BP measurement includes ambulatory BP monitoring and home BP monitoring, which can provide large amount of BP data outside the medical environment [30, 31]. In this trial, we will use clinic BP measurement to assess the variation in SBP and DBP from baseline to 12 months and use out-of-office BP measurement (home BP monitoring) to promote interventions in daily management. When performing clinic BP measurement, patients should be seated for at least 5 min in a quiet room before measurements and keep the upper arm at the heart level. Measurements will be conducted at least 2 times and taken 1–2 min apart. An additional measurement will be required in case that the first two readings differ by > 5 mmHg. The mean value of these readings will be recorded.

#### Biochemical indexes

Biochemical indexes are collected from the blood test, as shown in Table 2. These indexes are associated with cardiovascular risk factors, target organ damage, and the corresponding clinical conditions [32], which can indirectly reflect the control of hypertension.

#### Lifestyles

Lifestyle measure contains 3 parts: obesity, physical activity, and diet. Obesity is estimated by body mass index (BMI) and waist-to-hip ratio (WHR, for abdominal obesity), which will be measured in the basic body measurement. Physical activity will be assessed by the short form of International Physical Activity Questionnaire (IPAQ) [33]. Diet is evaluated by the intake of salt. Low-sodium diets will be assessed by the 24-h urine sodium collected from the 24-h urine test. The 24-h measurement of salt excretion in urine has proved to be the best method to measure salt intake [34]. Twenty-four-hour urine potassium will also be considered since increasing potassium intake properly in the diet can help reduce BP [35].

#### Self-management adherence

Adherence to self-management consists of 2 parts: medication adherence and self-monitoring adherence. Medication adherence will be assessed by the Hill-Bone Medication Adherence Scale (HB-MAS) including 9 items [36]. Self-monitoring adherence estimates the compliance with non-pharmacological treatment, which will be assessed by the system log data. We calculate the adherence based on the ratio of actual frequency of BP measurements to the number required by the management plan. The concrete formula can be found in our previous paper [28].

#### Hypertension awareness

Patients’ hypertension awareness reflects their self-management ability, which will be assessed by the Hypertension Knowledge-Level Scale (HK-LS) [37]. HK-LS contains 22 items with 6 sub-dimensions: definition, medical treatment, drug compliance, lifestyle, diet, and complication.

#### Work efficiency of care providers

Work efficiency of care providers will be calculated based on the follow-up data. In this trial, all the follow-ups are generated, conducted, and recorded through the system. The generated follow-up requests can be divided into 3 types: regular follow-up, abnormal attention follow-up, and compliance follow-up. The system needs to schedule all the follow-ups reasonably to make care providers handle requests timely. We propose 2 indexes to assess the work efficiency: one is the frequency of follow-up in a day, which demonstrates how our system reduces the time cost of a single follow-up; another one is the handling time of a follow-up request, which means the time duration before a generated follow-up request is handled. Intuitively, the more follow-up care providers conduct in a day and the less handling time a follow-up request costs, the more effective their work will be. We will derivate the average work efficiency of care providers in the entire trial period for the control group and the intervention group, respectively. For the handling time of a follow-up request, we will calculate time cost for the 3 types of follow-ups respectively, then sum it together with different weights to get the average time cost.

### Plans to promote participant retention and complete follow-up {18b}

At each assessment point (T1 and T2), all patients will receive a phone call from CMs to remind them of the upcoming data collection and free offline consultation with doctors if they attend the assessment. Patients who prematurely discontinue from the study before the 6-month evaluation and do not attend any assessment will have the following evaluations performed, if possible: First, GPs will call them to invite them to insist on the trial or attend the assessment; if refused, a brief interview will be conducted to investigate the reasons for discontinuation. Second, the system log data of these patients will be fully explored to extract useful information.

### Data management {19}

In this trial, most of the data during the management process will be recorded by the system and saved in the database. The assessment data will be entered electronically by an investigator. Double data entry will be performed, and all data ranges will be checked again by a different investigator. All patients, physicians, CMs, and GPs have their unique IDs, and their login passwords are encrypted and kept anonymous to the database administrator. The database is also password protected, with access only to the IPs which have been previously registered in a white list. The investigators are only provided with read permission to the database.

### Confidentiality {27}

All records that contain personal information will be stored separately in the database. Only care providers can view the information to conduct the intervention. In data analysis, all data will be treated anonymously. Participants’ personal information will not be released outside of the study without the written permission.

### Plans for collection, laboratory evaluation, and storage of biological specimens for genetic or molecular analysis in this trial/future use {33}

In this trial, a blood collection and a 24-h urine collection will be carried out in HMC for each participating patient at T0, T1, and T2. The blood and urine samples will be used for laboratory tests to assess the biochemical indexes related to hypertension (see Table 2). The test results will be sent to investigators and all samples will not be stored. Investigators will not have access to personal identifiers.

## Statistical methods

### Statistical methods for primary and secondary outcomes {20a}

Statistical analysis will be performed using SPSS version 23.0. All statistical tests will be reported as a two-sided significance level of 5%, and the results will be presented with two-sided 95% confidence intervals (CIs). The data will be treated either by parametric or non-parametric analysis based on the tests for normal distribution (Shapiro-Wilk test) and homoscedasticity (Levene’s test). We will first conduct detailed exploratory descriptive analyses to collected data using graphical and numerical summaries. Then, comparisons between and within groups will be performed to identify the effectiveness. For categorical variables, the Pearson chi-square test or Fisher exact test will be used; for continuous variables, the Student *t* test or Mann-Whitney test will be used. In case of significant baseline differences, a linear mixed model will be constructed for the outcomes adjusting the baseline using the following covariates as appropriate: baseline SBP, demographics (age, gender, education levels), and BMI. Only those confounding factors found to be statistically significant to the outcomes by analysis of covariance will be included in the final model.

### Interim analyses {21b}

This is not applicable. No formal interim analysis of the primary and secondary outcomes is planned.

### Methods for additional analyses (e.g., subgroup analyses) {20b}

We will also perform subgroup analyses of the primary and secondary outcomes stratified by following variates at the initial management (1 week after baseline): BMI (< 24, ≥ 24), management level (level 1 and level 2), and self-management adherence.

### Methods in analysis to handle protocol non-adherence and any statistical methods to handle missing data {20c}

The statistical analysis will be conducted on an intention-to-treat (ITT) basis. For incomplete assessments (missing data), multiple imputation will be applied using both an optimistic and pessimistic scenario.

### Plans to give access to the full protocol, participant-level data, and statistical code {31c}

The participant-level dataset and statistical code generated during the current study will be available from the corresponding author on reasonable request after the trial is finished.

## Oversight and monitoring

### Composition of the coordinating center and trial steering committee {5d}

Two committees have been established to manage the trial, including a trial steering committee and a data monitoring committee. The steering committee composes of cardiologists from the First People’s Hospital of Yibin and investigators from Zhejiang University. The major responsibility of the steering committee is to evaluate and study protocol, communicate with the ethics committee, and supervise the practice of hypertension management for both 2 groups.

### Composition of the data monitoring committee, its role and reporting structure {21a}

The data monitoring committee consists of statisticians from Zhejiang University. This committee is independent from the sponsor and has no competing interests. The committee will be in charge of auditing the trial every quarter to oversee the data quality of the study. The committee charter is available from the corresponding author.

### Adverse event reporting and harms {22}

Serious adverse events will be collected and recorded for further analysis at the end of the trial. These events include deaths, emergency room visits, and hospitalization longer than 24 h. Investigators will explore the correlation between these events and patients’ healthcare records.

### Frequency and plans for auditing trial conduct {23}

The trial conduct will be audited by the data monitoring committee once every quarter.

### Plans for communicating important protocol amendments to relevant parties (e.g., trial participants, ethical committees) {25}

All changes to the study protocol will be reviewed by the steering committee, then reported to the sponsor, participating care providers, and investigators.

## Dissemination plans {31a}

The study results will be disseminated via articles published in peer-reviewed journals.

## Discussion

This protocol has been designed to understand the effectiveness of a pathway-driveneHealth-based integrated care model, implemented as a full-featured telehealth system. Compared with prior studies [38, 39], our study innovatively introduces the concept of pathway, then combines it with information technology and the “three-manager” mode to establish a standardized and executable care model. Besides testing efficacy of the model, this study will have several other strengths if successful. First, the model can be generalized to other common chronic diseases, such as diabetes, stroke, and chronic obstructive pulmonary disease (COPD). The task-based pathway can be applied in both single disease and multiple chronic conditions. Second, the study would provide the feasibility evidence of the “three-manager” mode combined with information technology in China. Policy makers in other regions across the country could roll out similar services for better management. Last, the awareness, treatment, and control rates of hypertension in Sichuan, Yunnan, and Guizhou are relatively low compared with other provinces [40]. Therefore, performing such a trial in Yibin is significant to improve the local outcome for hypertension management.

The present protocol is also subject to some limitations. First, since the clients for patients are different from traditional mobile applications, participants may show different levels of acceptance in the system. Low system acceptance will lead to undesirable high attrition rates. We will adopt several strategies to improve patients’ acceptance of the system, including ongoing system updates with usability tests, online system guidance from CMs, and regular offline training sessions for patients. Second, this is an open-label trial for both patients and care providers. Patients in the control group may not comply with self-management regimes or even choose to discontinue the participation, and care providers may tend to conduct the management for the control group not following the guidelines. We plan to eliminate these potential biases through intensive training of care providers and reward mechanism on patients (for example, free outpatient registration), as well as supervision of intervention quality from the steering committee.

### Trial status

This study protocol was approved on 7 November 2019, and this manuscript details the protocol on the original version. Due to the COVID-19 pandemic, the participant recruitment has been adjusted to July 2020. The first participant was enrolled on July 3, 2020, and recruitment is expected to be completed by November 2020.

## Supplementary Information

**Additional file 1.** SPIRIT Checklist.

**Additional file 2.** TIDieR checklist.

## Abbreviations

BP: Blood pressure
CMs: Case managers
GPs: General practitioners
HB-MAS: The Hill-Bone Medication Adherence Scale
HK-LS: The Hypertension Knowledge-Level Scale
IPAQ: The International Physical Activity Questionnaire
PEICM: Pathway-driven eHealth-based integrated care model
