# Supplementary material for: Effectiveness of a pathway-driven eHealth-based integrated care model (PEICM) for community-based hypertension management in China: study protocol for a randomized controlled trial
Source: Trials. 2021 Jan 22;22:81. doi: 10.1186/s13063-021-05020-2 (PMC7820518; doi:10.1186/s13063-021-05020-2)
Supplement: Supplementary file 2 — Additional file 2. TIDieR checklist. [file 13063_2021_5020_MOESM2_ESM.docx]

# Reporting checklist for medical interventions.

Based on the TIDieR guidelines.

## Instructions to authors

Complete this checklist by entering the page numbers from your manuscript where readers will find each of the items listed below.

Your article may not currently address all the items on the checklist. Please modify your text to include the missing information. If you are certain that an item does not apply, please write "n/a" and provide a short explanation.

Upload your completed checklist as an extra file when you submit to a journal.

In your methods section, say that you used the TIDieRreporting guidelines, and cite them as:

Hoffmann T, Glasziou P, Boutron I, Milne R, Perera R, Moher D, Altman D, Barbour V, Macdonald H, Johnston M, Lamb S, Dixon-Woods M, McCulloch P, Wyatt J, Chan A, Michie S. Better reporting of interventions: template for intervention description and replication (TIDieR) checklist and guide. BMJ. 2014;348:g1687.

|  |  | Reporting Item | Page Number |
| --- | --- | --- | --- |
| **Methods** |  |  |  |
| Brief name | [#1](https://www.goodreports.org/tidier/info/#1) | Provide the name or a phrase that describes the intervention | 5-6 |
| Why | [#2](https://www.goodreports.org/tidier/info/#2) | Describe any rationale, theory, or goal of the elements essential to the intervention | 3-4 |
| What (materials) | [#3](https://www.goodreports.org/tidier/info/#3) | Describe any physical or informational materials used in the intervention, including those provided to participants or used in intervention delivery or in training of intervention providers. Provide information on where the materials can be accessed (for example, online appendix, URL) | 7 |
| What (procedures) | [#4](https://www.goodreports.org/tidier/info/#4) | Describe each of the procedures, activities, and/or processes used in the intervention, including any enabling or support activities. | 5-7 |
| Who provided | [#5](https://www.goodreports.org/tidier/info/#5) | For each category of intervention provider (for example, psychologist, nursing assistant), describe their expertise, background and any specific training given | 5-7 |
| How | [#6](https://www.goodreports.org/tidier/info/#6) | Describe the modes of delivery (such as face to face or by some other mechanism, such as internet or telephone) of the intervention and whether it was provided individually or in a group. | 5-7 |
| Where | [#7](https://www.goodreports.org/tidier/info/#7) | Describe the type(s) of location(s) where the intervention occurred, including any necessary infrastructure or relevant features | 4-5 |
| When and how much | [#8](https://www.goodreports.org/tidier/info/#8) | Describe the number of times the intervention was delivered and over what period of time including the number of sessions, their schedule, and their duration, intensity or dose | 5-7 |
| Tailoring | [#9](https://www.goodreports.org/tidier/info/#9) | If the intervention was planned to be personalised, titrated or adapted, then describe what, why, when, and how | n/a |
| Modifications | [#10](https://www.goodreports.org/tidier/info/#10) | If the intervention was modified during the course of the study, describe the changes (what, why, when, and how) | n/a |
| How well (planned) | [#11](https://www.goodreports.org/tidier/info/#11) | If intervention adherence or fidelity was assessed, describe how and by whom, and if any strategies were used to maintain or improve fidelity, describe them. | 8 |
| How well (actual) | [#12](https://www.goodreports.org/tidier/info/#12) | If intervention adherence or fidelity was assessed, describe the extent to which the intervention was delivered as planned. | n/a |

This checklist was completed on 4. February 2020 using <https://www.goodreports.org/>, a tool made by the [EQUATOR Network](https://www.equator-network.org) in collaboration with [Penelope.ai](https://www.penelope.ai)
